# Supplementary material for: Causal Relationship between Aging and Anorexia Nervosa: A White-Matter-Microstructure-Mediated Mendelian Randomization Analysis
Source: Biomedicines. 2024 Aug 16;12(8):1874. doi: 10.3390/biomedicines12081874 (PMC11351342; doi:10.3390/biomedicines12081874)
Supplement: Supplementary file 1 [file biomedicines-12-01874-s001.zip › Supplementary Figure/Figure S8/Leaveoneout plot of MR analysis for TL to ubm-b-1930.pdf]

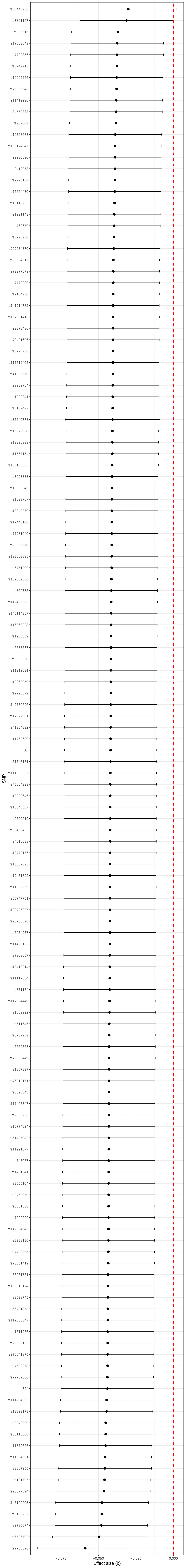
rs35446936
rs3891167
rs939916
rs17803849
rs7790856
rs5742915
rs10905255
rs76065543
rs11412296
rs34550383
rs932002
rs10768683
rs185174247
rs2230590
rs9419958
rs2276182
rs75664430
rs10112752
rs1291143
rs762679
rs6790988
rs202034370
rs80324517
rs79977579
rs7772289
rs7164950
rs141214782
rs137901416
rs9878436
rs78491606
rs6776756
rs117512405
rs41269079
rs2282764
rs1332941
rs8102497
rs35640778
rs16978028
rs12925933
rs11557154
rs150150565
rs3093888
rs10805346
rs1023767
rs10840270
rs17445108
rs77231040
rs28363070
rs139669835
rs6751209
rs182059586
rs869785
rs142426306
rs145114957
rs116863223
rs1985369
rs6587577
rs9955360
rs11212631
rs12369950
rs2293579
rs142730696
rs17677991
rs41304832
rs11769630
All
rs61748181
rs111950327
rs45604339
rs13230646
rs10845387
rs9600019
rs59409453
rs4616688
rs10773176
rs13062095
rs12451892
rs11699829
rs55747751
rs139795227
rs73730598
rs6054257
rs11426156
rs7209057
rs12412214
rs11117354
rs871134
rs117034449
rs1003322
rs611646
rs3767952
rs6669563
rs76666449
rs1957937
rs76219171
rs6590343
rs117407747
rs2056726
rs10774624
rs61405042
rs11991877
rs4743037
rs4731541
rs2555104
rs2763979
rs6881568
rs7099229
rs112394943
rs9398196
rs4498805
rs73581419
rs56061761
rs188918174
rs2538745
rs66731853
rs117630647
rs1611236
rs28502153
rs376641875
rs4530278
rs77732866
rs4724
rs144204502
rs12932179
rs9940099
rs80116508
rs11579626
rs11584821
rs2967355
rs131797
rs28577594
rs143190905
rs8105767
rs3785074
rs6536702
rs7705526
SNP
−0.075
−0.050
−0.025
0.000
Effect size (b)
